# From elementary flux modes to elementary flux vectors: Metabolic pathway analysis with arbitrary linear flux constraints

**DOI:** 10.1371/journal.pcbi.1005409

**Published:** 2017-04-13

**Authors:** Steffen Klamt, Georg Regensburger, Matthias P. Gerstl, Christian Jungreuthmayer, Stefan Schuster, Radhakrishnan Mahadevan, Jürgen Zanghellini, Stefan Müller

**Affiliations:** 1 Max Planck Institute for Dynamics of Complex Technical Systems, Magdeburg, Germany; 2 Institute for Algebra, Johannes Kepler University Linz (JKU), Linz, Austria; 3 Department of Biotechnology, University of Natural Resources and Life Sciences, Vienna, Austria; 4 Austrian Centre of Biotechnology, Vienna, Austria; 5 TGM - Technologisches Gewerbemuseum, Vienna, Austria; 6 Department of Bioinformatics, Faculty of Biology and Pharmacy, Friedrich Schiller University Jena, Jena, Germany; 7 Department of Chemical Engineering & Applied Chemistry, Institute of Biomaterials and Biomedical Engineering, University of Toronto, Toronto, Ontario, Canada; 8 Radon Institute for Computational and Applied Mathematics (RICAM), Austrian Academy of Sciences, Linz, Austria; Christian Albrechts Universitat zu Kiel, GERMANY

## Abstract

Elementary flux modes (EFMs) emerged as a formal concept to describe metabolic pathways and have become an established tool for constraint-based modeling and metabolic network analysis. EFMs are characteristic (support-minimal) vectors of the flux cone that contains all feasible steady-state flux vectors of a given metabolic network. EFMs account for (homogeneous) linear constraints arising from reaction irreversibilities and the assumption of steady state; however, other (inhomogeneous) linear constraints, such as minimal and maximal reaction rates frequently used by other constraint-based techniques (such as flux balance analysis [FBA]), cannot be directly integrated. These additional constraints further restrict the space of feasible flux vectors and turn the flux cone into a general flux polyhedron in which the concept of EFMs is not directly applicable anymore. For this reason, there has been a conceptual gap between EFM-based (pathway) analysis methods and linear optimization (FBA) techniques, as they operate on different geometric objects. One approach to overcome these limitations was proposed ten years ago and is based on the concept of *elementary flux vectors* (EFVs). Only recently has the community started to recognize the potential of EFVs for metabolic network analysis. In fact, EFVs exactly represent the conceptual development required to generalize the idea of EFMs from flux cones to flux polyhedra. This work aims to present a concise theoretical and practical introduction to EFVs that is accessible to a broad audience. We highlight the close relationship between EFMs and EFVs and demonstrate that almost all applications of EFMs (in flux cones) are possible for EFVs (in flux polyhedra) as well. In fact, certain properties can only be studied with EFVs. Thus, we conclude that EFVs provide a powerful and unifying framework for constraint-based modeling of metabolic networks.

## Introduction

Over the last 25 years, stoichiometric and constraint-based modeling (CBM) has emerged as a fundamental computational framework to analyze properties and capabilities of metabolic networks or to identify suitable targets for rational metabolic engineering [1,2,3,4,5,6]. Numerous CBM-based studies of realistic (up to genome-scale) metabolic networks demonstrated the broad applicability and acceptance of the methodology. The evolution of CBM techniques was driven by the goal to find appropriate mathematical formalisms to describe and analyze metabolic networks, in particular, in the case of insufficient knowledge of kinetic data. Due to their nature, CBM-related formalisms and methods are based on techniques from certain mathematical disciplines, including linear algebra, linear programming, convex analysis, and computational geometry. In fact, in some cases, CBM enforced the development of new theoretical concepts (if a suitable mathematical framework was not yet available) and of new algorithmic approaches to speedup computations in large metabolic network models. Elementary flux modes (EFMs), introduced by Schuster and Hilgetag in 1994 [7], represent one such example. EFMs arose as a conceptual development to describe metabolic pathways and to characterize the space of feasible steady-state flux distributions in metabolic networks. Various applications of EFMs proved the value of this approach [3,8,9,10]. Moreover, over the last 15 years, the scientific community achieved remarkable algorithmic developments, which speedup the computation of EFMs by several orders of magnitude [11,12,13,14,15,16], now outperforming standard implementations from computational geometry.

EFMs explicitly account for steady state and known reaction irreversibilities. However, other linear constraints such as minimal and maximal reaction rates, which are frequently used by many CBM techniques, cannot be directly integrated into EFM analysis. These additional constraints further restrict the space of steady-state flux distributions in the network and change it from a polyhedral cone to a general polyhedron. Differences in the shape and nature of these spaces hampered a clear correspondence between EFM-based pathway analysis methods and optimization-based methods such as flux balance analysis (FBA) [17]. Thus, there was a need to generalize the concept of EFMs to also characterize polyhedral spaces arising from arbitrary linear constraints on the network fluxes. It was only in 2007, 13 years after the first paper on EFMs, that Urbanczik [18] presented such an approach by introducing the concept of *elementary flux vectors* (EFVs). It took several more years until the community started to recognize the potential of EFVs, in particular, that they represent exactly the conceptual development required to generalize the idea of EFMs from flux cones to flux polyhedra. A recent publication demonstrated how EFVs can be used in a realistic application (for metabolic engineering purposes) [19], whereas another paper discussed theoretical properties of elementary vectors in the context of polyhedral geometry [20].

This work aims (i) to provide a motivation and profound introduction to EFVs; (ii) to explain important theoretical properties of EFVs; (iii) to discuss similarities and differences between EFMs and EFVs; and (iv) to highlight applications of EFVs that were not possible with existing techniques.

## Flux cones

In a minimal description, a metabolic network is given by (i) the stoichiometric matrix ***N*** ∈ ℝ^*m×n*^ containing the net stoichiometric coefficients of *m* internal metabolites in *n* reactions and (ii) irreversibility constraints for certain reactions. The steady—state assumption (concentrations of internal metabolites do not change) leads to the fundamental equation

Nr=0, (1)

where ***r*** is the *n*-dimensional vector of net reaction rates, also called rate vector, flux vector, or flux distribution. Only if Eq 1 is fulfilled, consumption and production of each intracellular metabolite over all reactions in the network will be balanced as required for steady state. The set of flux vectors ***r*** satisfying Eq 1 is the *nullspace* of ***N***, having dimension *n* − rank(***N***). Irreversible reactions, contained in the set *Irr*, have sign restrictions on their rates which can be expressed by the inequalities

$$r_{i}\geq 0\;\;\text{for }i\in Irr.$$ (2)

The right-hand sides of Eqs 1 and 2 contain only zeros and are therefore *homogeneous constraints* on the flux vectors ***r***. The set *FC* of flux vectors ***r*** satisfying Eqs 1 and 2

$$FC=\left\{r\in {\mathbb{R}}^{n}|Nr=0,\;r_{i}\geq 0\;\text{for }i\in Irr\right\},$$ (3)

is a subset of the nullspace of ***N***. More precisely, it is the intersection of the nullspace with the nonnegative halfspaces corresponding to the irreversible reactions. In geometrical terms, this set is a *convex polyhedral cone*, which, in the context of metabolic network analysis, is called the *flux cone* (denoted here by *FC*).

**Fig 1:**
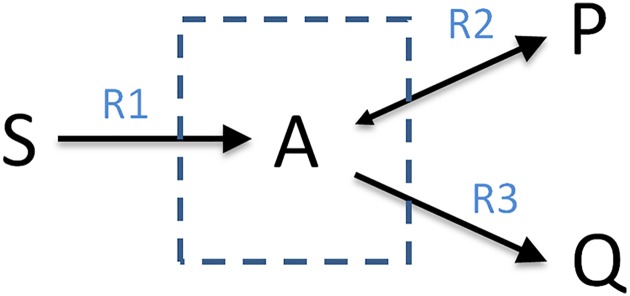
A simple example network.

**Fig 2:**
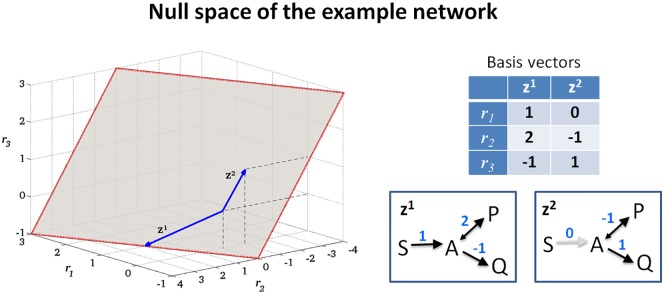
The nullspace of the example network. The red lines indicate that the nullspace is unbounded (in all directions). The blue arrows are basis vectors, which are also depicted as flux distributions.

**Fig 3:**
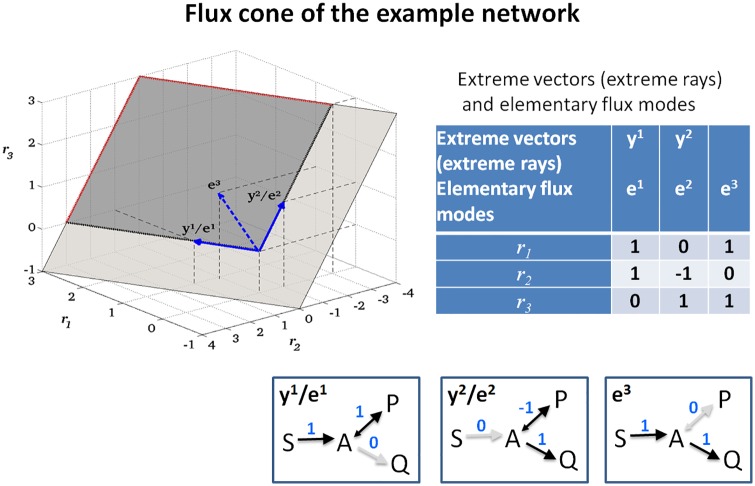
The flux cone of the example network. The flux cone (dark grey) is obtained from the nullspace in Fig 2 by removing the halfspaces corresponding to the backward directions of the irreversible reactions R1 and R3 (removed part shown in light grey). The red dotted lines indicate the unbounded directions of the cone. The cone is bounded by its extreme rays (bold black dotted lines), which are represented by the extreme vectors ***y***^1^ and ***y***^2^(full blue arrows). Both extreme vectors are also elementary flux modes, and a third elementary flux mode exists in the interior of the cone (dashed blue arrow). The extreme vectors and elementary flux modes, respectively, are also depicted as flux distributions. Note that the flux cone is unbounded, however, it appears as a rectangle because it is cropped by the bounding box.

**Example**: To illustrate the theoretical concepts, we consider a running example, namely the minimal network shown in Fig 1. Although very simple, a similar network was recently used to model the Warburg and the Crabtree effect [21,22]. It consists of one internal metabolite (A), three external metabolites (S, P, Q), and three reactions, where reactions R1 and R3 are assumed to be irreversible. The corresponding stoichiometric matrix amounts to ***N*** = (1, −1, −1), and the index set of irreversible reactions is given by *Irr* = {1,3}. Fig 2 shows the nullspace {***r*** ∈ ℝ^3^ | ***Nr*** = **0**}, having dimension *n* − rank(***N***) = 3 − 1 = 2. Thus, any two linearly independent vectors fulfilling Eq 1 form a basis of the nullspace; for example, we may choose the basis ***z***^1^ = (1,2,−1)^*T*^ and ***z***^2^ = (0,−1,1)^*T*^. Note that these vectors are a valid basis of the nullspace but ***z***^1^ violates the sign restrictions of Eq 2. Fig 3 shows the flux cone *FC* = {***r*** ∈ ℝ^3^ | ***Nr*** = **0** and *r*_1_,*r*_3_ ≥ 0} of the example network. As can be seen, the flux cone is, by definition, a subset of the nullspace, and the vector ***z***^1^ = (1,2,−1)^*T*^ is not contained in the flux cone as it violates the sign restriction for reaction R3.

As mentioned above, the flux cone is a special polyhedral cone. A *general polyhedral cone* *C* can be represented as

$$C=\left\{x\in {\mathbb{R}}^{n}|Ax\geq 0\right\}$$ (4)

with a suitable matrix ***A*** ∈ ℝ^*q*×*n*^ and is thus an intersection of *q* halfspaces passing through the origin. Flux cones are special as they are contained in a lower-dimensional subspace (the nullspace) and involve only equality and nonnegativity constraints (see also [20]). Still, the flux cone (Eq 3) can be written in the general form (Eq 4) by setting

$$A=\left(\begin{array}{c} N\\ -N\\ I_{Irr} \end{array}\right),$$

where ***I***_*Irr*_ ∈ ℝ^|*Irr*|×*n*^ is a matrix representing the nonnegativity constraints (and |*Irr*| is the number of irreversible reactions). In the example, we have

$$A=\left(\begin{array}{ccc} 1 & -1 & -1\\ -1 & 1 & 1\\ 1 & 0 & 0\\ 0 & 0 & 1 \end{array}\right).$$

As a key property of cones, for every nonzero element ***x*** of a polyhedral cone *C*, the whole *ray* {*β****x*** | *β* ≥ 0} is contained in the cone. A cone may have a nontrivial *lineality space* containing all vectors ***x*** ∈ *C* for which also −***x*** ∈ *C*. In other words, the lineality space contains all lines of the cone. Note that the lineality space of a cone *C*, as given by Eq 4, equals the nullspace of ***A***, that is, {***x*** ∈ ℝ^*n*^ | ***Ax* = 0**}. A cone is *pointed* if it does not contain a line, that is, if its lineality space is trivial (contains the zero vector only). The lineality space has important consequences for the geometry and the generators (see below) of a cone. If a cone is pointed, then the zero vector is the only vertex (*extreme point*) of the cone, whereas a cone with nontrivial lineality space does not have a vertex at all. A *vertex* cannot be written as a convex linear combination of other vectors (see below).

The lineality space contains reversible steady-state flux vectors. In general, chemical reactions are reversible, and the lineality space of a network can be nontrivial. In this case, there are flux vectors which use only reversible reactions (and have zero entries for irreversible reactions). In metabolic networks under typical biological conditions, many reactions are practically irreversible and reversible flux vectors occur only rarely in metabolic network models (in fact, opposite metabolic pathways such as glycolysis and gluconeogenesis often use slightly different routes for thermodynamic reasons). In other words, metabolic network models often give rise to pointed flux cones.

**Example**: In our example in Fig 1, there is no reversible flux vector (the nullspace of ***A*** is trivial) and hence the flux cone is pointed (Fig 3).

As an alternative to the so-called *implicit* representation (Eq 4), every polyhedral cone can be written *explicitly* as a sum of finitely many generators. More precisely, as a *conical* (nonnegative linear) *combination* of vectors ***y***^*j*^ ∈ ℝ^*n*^ (*j* ∈ *J*), which are not in the lineality space, plus a *linear combination* of vectors ***z***^*k*^ ∈ ℝ^*n*^ (*k* ∈ *K*) of the lineality space, where *J* and *K* are finite index sets [23,24,25]:

$$C=\left\{\Sigma_{j\in J}\;\beta_{j}y^{j}+\Sigma_{k\in K}\;\gamma_{k}z^{k}|\beta_{j}\geq 0,\;\gamma_{k}\in \mathbb{R}\right\}.$$ (5)

However, the set of *generators* ***y***^*j*^ and ***z***^*k*^ is not unique. In fact, not even a minimal set of generators (with smallest number of generators) is unique. Only if the cone is pointed (the lineality space is trivial), there exists a unique minimal set of generators. In this case, generators ***z***^*k*^ of the lineality space are not needed and the generators ***y***^*j*^ are unique (up to positive scalar multiplication); they are a set of *extreme vectors* of the pointed cone, each representing an extreme ray of the cone. Formally, a nonzero vector ***x*** ∈ *C* is *extreme* if it cannot be written as a sum ***x*** = ***x***^1^ + ***x***^2^ of linearly independent vectors ***x***^1^,***x***^2^ ∈ *C*. The corresponding extreme ray is given by {*β****x*** | *β* ≥ 0}. Geometrically, extreme rays are the “edges” of a pointed cone.

For nonpointed cones (cones with nontrivial lineality space), the situation is more involved. In particular, extreme rays do not exist. A (nonunique) minimal set of generators consists of suitable vectors ***y***^*j*^ and basis vectors ***z***^*k*^ of the lineality space. (Regarding the vectors ***y***^*j*^, one may choose, for example, the extreme vectors of a pointed cone that is obtained by intersecting the original cone with the [orthogonal] complement of its lineality space [23].)

**Example**: In our running example (Fig 1), the flux cone is pointed and generated by the two extreme vectors ***y***^1^ = (1,1,0)^*T*^ and ***y***^2^ = (0,−1,1)^*T*^, each representing an extreme ray (see Fig 3). If we set reaction R3 reversible, then the flux cone is not pointed anymore. The lineality space would then involve the reversible reactions R2 and R3 and could be described by the single basis vector (0,−1,1)^*T*^. For the generators in Eq 5, we could then choose ***y***^1^ = (2,1,1)^*T*^ (from the orthogonal complement of the lineality space) and ***z***^1^ = (0,−1,1)^*T*^, a basis vector of the lineality space. Note that we could replace ***y***^1^, for instance, by ***y***^1^ + ***z***^1^ = (2,0,2)^*T*^, indicating the nonuniqueness of the minimal set of generators.

In metabolic pathway analysis, one is often interested in minimal pathways and cycles (with as few reactions as possible operating at steady state) to characterize the flux cone. In mathematical terms, minimal pathways are support-minimal vectors of the flux cone. Formally, a nonzero flux vector ***r*** ∈ *FC* is *support minimal* if there is no nonzero vector ***r′*** ∈ *FC* such that *supp*(***r***′) ⊂ *supp*(***r***), where *supp*(***r***) = {*i* | *r*_*i*_ ≠ 0} and ⊂ indicates a proper subset. The question arises whether extreme vectors are such minimal pathways. In fact, in pointed *flux* cones *FC*, the extreme vectors (forming the minimal set of generators) are indeed always support minimal (which is not true for general pointed cones *C*). However, even in pointed flux cones, the set of extreme vectors need not cover *all* support-minimal flux vectors.

**Example**: In our example network, S→P, S→Q, and P→Q are the minimal pathways represented by the support-minimal vectors ***e***^1^ = (1,1,0)^*T*^, ***e***^2^ = (0,−1,1)^*T*^, and ***e***^3^ = (1,0,1)^*T*^, respectively (Fig 3). In fact, ***e***^1^ and ***e***^2^ coincide with the extreme vectors ***y***^1^ and ***y***^2^, whereas ***e***^3^ = ***e***^1^ + ***e***^2^ is not an extreme vector, because it can be written as a sum of ***e***^1^ and ***e***^2^. Hence, the pathway S→Q would clearly be missed when restricting the analysis to the minimal set of generators given by the extreme vectors.

We note that extreme vectors of flux cones have been proposed as alternative concepts for metabolic pathway analysis, cf. *extreme currents* and *extreme pathways* [6,26]. For a comparison of the different concepts, see [27,28,29,30].

## Elementary (flux) modes

As stated above, the minimal set of generators need not be unique and need not contain all support-minimal vectors of the flux cone. These facts were the main motivation to introduce the concept of *elementary flux modes* (EFMs): EFMs are defined as the nonzero, support-minimal vectors of the flux cone. That is, a nonzero vector ***e*** ∈ *FC* is an EFM, if there is no nonzero vector ***r*** ∈ *FC* such that

supp(r)⊂supp(e).

Clearly, if ***e*** ∈ *FC* is an EFM, then all nonzero elements of the ray {*β****e*** | *β* ≥ 0} are EFMs (having the same support as ***e*)**. Typically, one chooses one representative EFM on each ray. The set of representative EFMs is thus unique up to positive scalar multiplication. In addition to being support minimal, EFMs are often also described as *nondecomposable* [7] or *irreducible* vectors of the flux cone.

An EFM is called irreversible if its support contains at least one irreversible reaction (and hence can operate in only one direction). Otherwise, it is called reversible (and can run in both directions). Clearly, reversible EFMs exist if, and only if, the lineality space is nontrivial. In our example network, the pointed flux cone contains three irreversible EFMs ***e***^1^, ***e***^2^, and ***e***^3^, each representing one minimal pathway connecting external metabolites (Fig 3).

The defining property of EFMs (support minimality) implies three important theoretical properties which turned out to be extremely useful for metabolic network analysis:

- (T1) The set of EFMs generates the flux cone (i.e., EFMs form a set of generators): every element of the flux cone *FC* can be written as a conical (nonnegative) linear combination of EFMs. Formally, let ***e***^*j*^ (*j* ∈ *J*) be a set of representative EFMs. Then,

$$FC=\left\{\Sigma_{j\in J}\;\beta_{j}e^{j}|\beta_{j}\geq 0\right\}.$$ (6)

In particular, the set of EFMs contains the extreme vectors if the cone is pointed or a basis of the lineality space if the latter is nontrivial. However, the set of EFMs is usually not a minimal set of generators, cf. Fig 3. Importantly, if a (representative) EFM ***e*** is reversible, then also −***e*** is an EFM. In the sum in Eq 6, the term *β****e*** + *β*′(−***e***) with *β*, *β*′ ≥ 0 can then be replaced by *γ****e*** with *γ* ∈ ℝ, giving rise to a sum as in Eq 5.

- (T2) After deleting a set of reactions, the set of EFMs of the resulting subnetwork needs not be recalculated. It can be directly derived from the EFMs of the full network, namely as the subset of EFMs not involving the deleted reactions. In other words, the set of EFMs of the full network contains the sets of EFMs of all possible subnetworks.

**Example**: In our example (Fig 3), if we delete reaction R2, then the EFMs ***e***^1^ and ***e***^2^ get eliminated, while ***e***^3^ is the only remaining support-minimal vector. The resulting flux cone equals the ray determined by ***e***^3^. Note that a similar procedure is not possible with the shown extreme vectors ***y***^1^ and ***y***^2^ as both would get eliminated.

To formulate the third property, we introduce the concept of *conformality*. Let ***r***^1^,***r***^2^ ∈ *FC* be two flux vectors. The sum ***r*** = ***r***^1^ + ***r***^2^ is called *conformal* if *r*_*i*_ = 0 implies $r_{i}^{1}=r_{i}^{2}=0$, *r*_*i*_ > 0 implies $r_{i}^{1},r_{i}^{2}\geq 0$, and *r*_*i*_ < 0 implies $r_{i}^{1},r_{i}^{2}\leq 0$ for all *i* ∈ {1, …, *n*}. In other words, a conformal sum does not involve *cancellations*.

**Example**: In our example, ***e***^3^ = ***e***^1^ + ***e***^2^ is not a conformal sum, because $e_{2}^{1}=1$ and $e_{2}^{2}=-1$.

- (T3) Every element of the flux cone can be written as a sum of EFMs *without cancellations*. In fact, the set of EFMs is the unique minimal set of *conformal* generators of the flux cone [20].

As it turns out, EFMs can be defined as *conformally nondecomposable* vectors of the flux cone. In other words, an EFM cannot be written as a sum of other flux vectors of the flux cone without cancellations [20]. This definition immediately implies (T3) and further the other properties (T1) and (T2) stated above. As we will see later, the definition of EFMs as conformally non-decomposable vectors (but not the definition as support-minimal vectors) can be extended from flux cones to general polyhedral cones.

By definition, a flux vector is a vector of net reaction rates. Then, only a conformal decomposition of a flux vector (a decomposition without cancellations) is biochemically meaningful, because a chemical reaction cannot have a net rate in different directions in the contributing EFMs. Thus, conformality accounts for a fundamental constraint arising from the second law of thermodynamics, which states that a reaction can only carry flux in the direction of negative Gibbs free energy of reaction.

## Applications of EFMs

EFMs have become a standard tool for analyzing medium-scale metabolic networks (typically models of the central metabolism) in which their computation is feasible. Here, we list a selection of applications (see also [3,8,9]):

- (A1) Due to their support minimality, EFMs correspond to minimal subnetworks that perform a certain function at steady state and have therefore been used to identify minimal conversion routes (pathways) or cycles in metabolic networks.
- (A2) EFMs can be used to predict network properties such as gene/reaction essentialities, structural couplings, blocked reactions, etc.
- (A3) Using EFMs, all yield-optimal routes for product or biomass synthesis can be identified. Moreover, EFMs have been used to investigate metabolic trade-offs [31] and to characterize optimal solutions of enzyme allocation problems in metabolic networks with kinetic information [21].
- (A4) EFMs are frequently used to identify intervention strategies for a targeted modification (engineering) of metabolic networks [32,33,34], including techniques,such as minimal metabolic functionality [35], FluxDesign [36], or minimal cut sets [37,38]. Most of these methods make heavy use of EFM property (T2), which facilitates a directed search for suitable interventions by blocking undesired while preserving desired phenotypes.
- (A5) Several approaches have been presented in the literature to decompose a flux distribution into metabolic pathways (that is, EFMs) to identify the contribution of certain modules to a given metabolic phenotype (e.g., [39,40]).

EFMs have also been used for a network-based analysis of experimental data including gene expression data [41,42] or metabolome data to identify thermodynamic bottlenecks [43,44,45,46].

Some of the network properties listed above (including essentialities, coupled reactions, and maximum yields) can also be studied with dedicated optimization-based approaches (e.g., FBA or flux variability analysis [5]). However, when an explicit *enumeration* of (optimal) pathways, intervention strategies, etc. is needed, that is, when (all) alternative solutions have to be identified, the concept of EFMs becomes the method of choice.

## Inhomogeneous constraints and (flux) polyhedra

**Fig 4:**
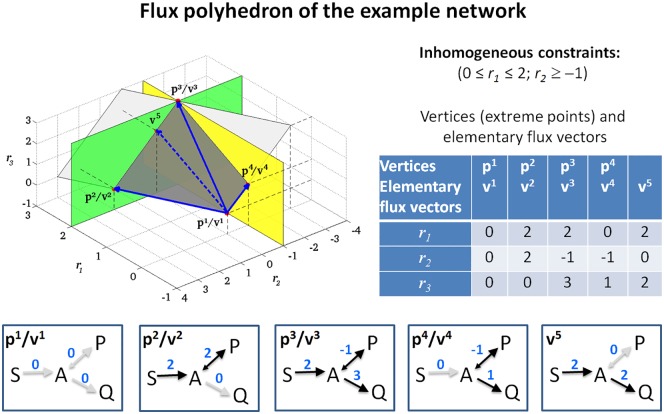
A bounded flux polyhedron in the example network. The two additional inhomogeneous constraints (an upper flux bound for reaction R1 and a lower flux bound for reaction R2) give rise to two hyperplanes *r*_1_ = 2 and *r*_2_ = −1 (green and yellow). These hyperplanes cut out the bounded flux polyhedron (dark grey) from the unbounded flux cone of Fig 3 (light grey). The polyhedron has five elementary flux vectors (full/dashed blue arrows and the zero vector), four of which correspond to vertices (full blue arrows and zero). The vertices and elementary flux vectors of the polyhedron are also depicted as flux distributions.

The concept of EFMs can be applied as long as we operate on the flux cone *FC*, generated by the two homogeneous constraints in Eqs 1 and 2. However, further linear constraints are frequently used to confine the set of feasible fluxes. This includes, in particular, lower (lb) and upper (ub) flux bounds,

$$r_{i}^{\text{lb}}\leq r_{i}\leq r_{i}^{\text{ub}},$$

typically known for certain (exchange) reactions *i*. In our example, we add an upper flux bound for reaction R1(*r*_1_ ≤ 2) and a lower flux bound for reaction R2 (*r*_2_ ≥ −1); see Fig 4. Flux bounds and other linear constraints can be written in the general form

Gr≥h, (7)

For the example in Fig 4 we write

$$\left(\begin{array}{lll} -1 & 0 & 0\\ 0 & 1 & 0 \end{array}\right)r\geq \left(\begin{array}{c} -2\\ -1 \end{array}\right).$$

Equality constraints can also be integrated in Eq 7. For instance, we could be interested in the space of optimal solutions for a given objective function [25]. In our example, we might optimize the production of P and Q, that is, we maximize the objective function *r*_2_ + *r*_3_. Using FBA (with flux bounds as defined above), we would find that the maximum value is 2. For describing the optimal solution space, we add the equality constraint for the optimum; this equality constraint can again be represented by two corresponding inequality constraints and we obtain:

$$\left(\begin{array}{lll} -1 & 0 & 0\\ 0 & 1 & 0\\ 0 & 1 & 1\\ 0 & -1 & -1 \end{array}\right)r\geq \left(\begin{array}{c} -2\\ -1\\ \begin{array}{c} 2\\ -2 \end{array} \end{array}\right).$$ (8)

In contrast to the homogeneous inequality constraints (Eq 2) in the definition of the flux cone, the *inhomogeneous* constraints (Eq 7) are “game changers,” because the vector ***h*** has then nonzero entries, as in the examples above. The set *FP* of flux vectors ***r*** satisfying the homogeneous and inhomogeneous constraints (Eqs 1, 2 and 7),

$$FP=\left\{r\in {\mathbb{R}}^{n}|Nr=0,\;\;r_{i}\geq 0\;\text{for}\;i\in Irr,\;Gr\geq h\right\},$$ (9)

forms a (bounded or unbounded) *polyhedron*, which, in the context of metabolic network analysis, is called the *flux polyhedron*. Clearly, *FP* is a subset of the flux cone *FC* (Eq 3). Some EFMs of the flux cone may still be contained in the flux polyhedron, but, in general, not a single EFM might fulfill the additional constraints.

**Example**: In our example network, if we used the flux bounds *r*_1_ ≥ 2 and *r*_2_ ≤ 1, then only a part of the ray determined by the EFM ***e***^3^ = (1,0,1)^*T*^ is contained in the flux polyhedron (namely where *r*_1_ ≥ 2), whereas the entire rays determined by the EFMs ***e***^1^ = (1,1,0)^*T*^ and ***e***^2^ = (0,−1,1)^*T*^ do not fulfill the additional constraints. If we use instead the flux bounds *r*_1_ ≥ 2 and *r*_2_ ≤ −1, then none of the EFMs is contained in the flux polyhedron anymore.

Evidently, in general, EFMs of the flux cone cannot describe a flux polyhedron. For this reason, there has always been some gap between EFM analysis (operating on the flux cone) and linear optimization techniques such as FBA (operating on a flux polyhedron arising from additional inhomogeneous constraints) [47]. The key question is whether, similar to EFMs, designated vectors can be identified for a flux polyhedron, which share the key properties (T1)–(T3) and allow for applications (A1)–(A5). Ideally, in the special case of a flux cone, this set of vectors should coincide with the set of EFMs.

Similarly, as the flux cone is a special polyhedral cone, the flux polyhedron is a special polyhedron because it is contained in a lower-dimensional subspace (the nullspace of the stoichiometric matrix). A general polyhedron is given by

$$P=\left\{x\in {\mathbb{R}}^{n}|Ax\geq b\right\}$$ (10)

for some matrix ***A*** ∈ ℝ^*q*×*n*^ and some vector ***b*** ∈ ℝ^*q*^. That is, a polyhedron is an intersection of *q* (affine) halfspaces. The flux polyhedron (Eq 9) can also be written as in Eq 10 by setting

$$A=\left(\begin{array}{c} N\\ -N\\ I_{Irr}\\ G \end{array}\right)\;\text{and }b=\left(\begin{array}{c} 0\\ 0\\ 0\\ h \end{array}\right).$$ (11)

Polyhedral cones, as given by Eq 4, are special types of polyhedra where ***b*** = **0** in (10). Every polyhedron *P* has an associated polyhedral cone *C*_*P*_ = {***x*** ∈ ℝ^*r*^ | ***Ax*** ≥ **0**}, called the *recession cone*, which contains the (unbounded) directions of the polyhedron [23]. Most importantly, every polyhedron can be written as a bounded polyhedron (a *polytope*) plus its recession cone, and hence a polyhedron is unbounded whenever its recession cone is nontrivial. The associated polytope is a *convex* combination of finitely many “bounded" generators ***p***^*i*^ ∈ ℝ^*n*^ (*i* ∈ *I*), whereas the recession cone, analogous to Eq 5, is a combination of the “unbounded” generators ***y***^*j*^ ∈ ℝ^*n*^ (*j* ∈ *J*) and ***z***^*k*^ ∈ ℝ^*n*^ (*k* ∈ *K*):

$$P=\left\{\Sigma_{i\in I}\;\alpha_{i}p^{i}+\Sigma_{j\in J}\;\beta_{j}y^{j}+\Sigma_{k\in K}\;\gamma_{k}z^{k}|\alpha_{i},\beta_{j}\geq 0,\;\gamma_{k}\in \mathbb{R},\;\Sigma_{i\in I}\;\alpha_{i}=1\right\}.$$ (12)

The recession cone is crucial for properties and generators of the polyhedron. A polyhedron is pointed if its recession cone is pointed (has trivial lineality space) and thus no generators of type ***z***^*k*^ exist. As in the case of polyhedral cones, only if the polyhedron is pointed, there exists a unique minimal set of generators of the polyhedron. It is given by the unique set of extreme vectors ***y***^*j*^ (corresponding to the extreme rays) of the recession cone and the unique bounded generators, which are the vertices (extreme points) of the polyhedron. Formally, a vector ***x*** ∈ *P* is a *vertex* if it cannot be written as a *convex combination* ***x*** = *λ****x***^1^ + (1 − *λ*)***x***^2^ of distinct vectors ***x***^1^,***x***^2^ ∈ *P* with 0 < *λ* < 1. If the polyhedron is not pointed, it does not have vertices and the minimal set of generators is not unique, as in the case of cones.

We emphasize again that metabolic network models often give rise to pointed flux cones or *pointed flux polyhedra* where reversible flux vectors do not exist. Moreover, in many flux optimization studies, the recession cone of the flux polyhedron is even trivial (equals the zero vector) meaning that (unrealistic) unbounded flux vectors do not exist (see also [25]).

A bounded polyhedron has a trivial recession cone. In this case, no unbounded generators ***y***^*j*^ and ***z***^*k*^ exist in Eq 12, and the polyhedron is minimally generated by the set of its vertices. As already mentioned, a polyhedral cone is a special case of a polyhedron. In fact, the only vertex of a pointed cone is the zero vector, whereas a non-pointed cone does not have a vertex at all. In both cases, the first sum in Eq 12 yields the zero vector, hence, one obtains Eq 5.

**Example**: In our example with additional flux constraints *r*_1_ ≤ 2 and *r*_2_ ≥ −1, the resulting flux polyhedron is bounded (a polytope) because its recession cone is trivial, and no unbounded generators exist (Fig 4). It is minimally generated by its vertices ***p***^1^ = (0,0,0)^*T*^, ***p***^2^ = (2,2,0)^*T*^, ***p***^3^ = (2,−1,3)^*T*^, and ***p***^4^ = (0,−1,1)^*T*^.

The explicit representation (Eq 12) of a (flux) polyhedron using minimal generators is useful for some applications, but it comes with the same disadvantages as with the representation of flux cones in Eq 5: minimal generators usually do not satisfy the theoretical properties (T1)–(T3). In particular, support-minimal flux vectors need not be contained in a minimal set of generators, and hence deletion studies and target identification applications (A4) are not possible. This can also be seen in our example, in which the additional vector ***v***^5^ is support minimal, but not contained in the minimal set of generators (Fig 4). If we delete reaction R2 (the flux is set to zero), the vertices ***p***^2^, ***p***^3^, ***p***^4^ become infeasible, and no nonzero flux vector remains in the minimal set of generators. However, the additional generator ***v***^5^ = (2,0,2)^*T*^ is still operable and generates, together with ***p***^1^ = (0,0,0)^*T*^, the flux polyhedron of the resulting subnetwork.

We note that Kelk et al. [25] used minimal generators, as in Eq 12, to characterize the growth-rate optimal flux space (a flux polyhedron). Thereby, some important (e.g., support-minimal) flux vectors might be missed, as was later pointed out in [47]. In fact, as in the case of flux cones, we need another set of vectors to characterize flux polyhedra and we finally approach the concept of *elementary (flux) vectors*, which will take over the role of EFMs.

## Elementary (flux) vectors

Elementary vectors (EVs) of linear subspaces were introduced by Rockafellar [48], whereas EVs of general polyhedral cones (Eq 4) were first used by Urbanczik and Wagner to analyze so-called conversion cones of metabolic networks [49]. Later, Urbanczik extended the concept to general (flux) polyhedra by using the method of homogenization [18]. Here, we give a more direct definition: EVs of a polyhedron are obtained by intersecting the polyhedron with all (closed) orthants. The resulting subpolyhedra have unique minimal sets of generators and their union forms the set of EVs of the polyhedron.

More formally, an orthant *Ω*_***s***_ ∈ ℝ^*n*^ is defined by a sign pattern ***s*** ∈ {−1,1}^*n*^,

$$\Omega_{s}=\left\{x\in {\mathbb{R}}^{n}\;|\;x_{i}s_{i}\geq 0\text{ for }i=1,\ldots ,n\right\},$$

that is, by specifying a sign (direction) for every coordinate (reaction). Clearly, there are 2^*n*^ orthants in ℝ^*n*^. Given a polyhedron *P* and an orthant *Ω*_***s***_, we introduce the subpolyhedron *P*_*s*_ = *P* ∩ *Ω*_***s***_, it is pointed by definition, because all elements have a unique sign pattern ***s***. Hence, it is minimally generated by its vertices and extreme vectors (one for each extreme ray of its recession cone). The set of *EVs* of *P* is now defined as the union of the minimal generators of all subpolyhedra. Hence, by definition, the set of EVs consists of *bounded* EVs (the vertices of the subpolyhedra) and *unbounded* EVs (the extreme vectors of the subpolyhedra’s recession cones). The bounded EVs are unique, and the unbounded EVs are unique up to positive scalar multiplication.

**Fig 5:**
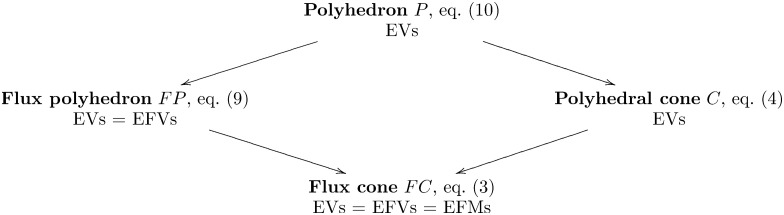
Relationships between (flux) polyhedra and (flux) cones and their Elementary Vectors (EVs)/Elementary Flux Vectors (EFVs)/Elementary Flux Modes (EFMs).

A flux polyhedron (Eq 9) is a special case of a general polyhedron (Eq 10), and its EVs are also called *elementary flux vectors* ((EFVs) [18]; see Fig 5. Further, a general polyhedral cone (Eq 4) is a special case of a polyhedron (Eq 10), and its EVs are the extreme vectors of the pointed subcones obtained when intersecting the cone with all closed orthants [49]. Finally, a flux cone (Eq 3) is a special case of both a flux polyhedron (Eq 9) and a general polyhedral cone (Eq 4), and its nonzero EFVs coincide with its EFMs (Fig 5). Hence, EVs (of polyhedra) generalize EFMs (of flux cones), and the three key theoretical properties of EFMs are preserved:

- (T1’) The set of EVs generates the polyhedron: every element of the polyhedron *P* can be written as a convex combination of bounded EVs plus a conical (nonnegative) linear combination of unbounded EVs. Formally, let ***v***^*i*^ (*i* ∈ *I*) and ***u***^*j*^ (*j* ∈ *J*) be the sets of bounded and unbounded EVs, respectively. Then,
  $$P=\left\{\Sigma_{i\in I}\;\alpha_{i}v^{i}+\Sigma_{j\in J}\;\beta_{j}u^{j}|\alpha_{i},\beta_{j}\geq 0,\;\Sigma_{i\in I}\;\alpha_{i}=1\right\}.$$ (13)
  In particular, the set of bounded EVs contains the set of vertices (for pointed polyhedra). However, as for EFMs, the set of EVs is not minimal (see also the example below).

If an unbounded EV ***u*** lies in the lineality space of the polyhedron, then also −***u*** is an unbounded EV. In the sum in Eq 13, the term *β****e*** + *β*′(−***e***) with *β*, *β*′ ≥ 0 can then be replaced by *γ****e*** with *γ* ∈ ℝ, and one obtains a sum of the form as in Eq 12.

We also note that, whenever the zero vector is contained in the polyhedron *P*, it is a vertex of all subpolyhedra *P*_*s*_ and thus constitutes an EV. While it is essential to keep the zero EV to generate the polyhedron, as in Eq 13, some applications exclude it (implicitly) from the analysis to focus on nontrivial flux distributions.

- (T2’) After deleting a set of reactions, the set of EVs/EFVs of the resulting subnetwork can be directly derived from the EFVs of the full network, namely as the subset of all EFVs not involving these reactions. In other words, the set of EFVs of the full network contains the sets of EFVs of all possible subnetworks. Thus, like EFMs, EFVs “anticipate” the deletion of any set of reactions and immediately provide a new set of generators after network modification.
- (T3’) Every element of a polyhedron can be written as a sum of EVs, as in Eq 13, *without cancellations*. In fact, the EVs form the unique minimal set of *conformal* generators of the polyhedron [20].

As it turns out, bounded EVs can be defined as convex-conformally nondecomposable vectors of the polyhedron, whereas unbounded EVs can be defined as conformally nondecomposable vectors of its recession cone [20]. That is, an EV cannot be written as a sum of other vectors of the polyhedron without cancellations.

Despite the shared key properties (T1)–(T3), there is also an important difference between EFMs and EVs/EFVs. As already mentioned above, a definition of EVs using support minimality (as for EFMs) is not possible. In particular, an EV need not be support minimal, as will be exemplified below. However, (T3’) immediately implies that, for every vector ***x*** ∈ *P*, there exists a (bounded or unbounded) EV ***v*** ∈ *P* with *supp*(***v***) ⊆ *supp*(***x***). In particular, if a vector ***x*** ∈ *P* has minimal support, there exists an EV ***v*** ∈ *P* with *supp*(***v***) = *supp*(***x***), hence, all minimal (support) patterns of flux vectors appear in the set of EVs.

**Example**: We return to our example (Fig 4). The flux polyhedron is bounded and has five (bounded) EFVs ***v***^1^, …, ***v***^5^. The first four EFVs coincide with the vertices ***p***^1^, …, ***p***^4^ of the polytope, which form the minimal set of generators. The additional EFV ***v***^5^ = (2,0,2)^*T*^ is not a vertex (and thus not part of the minimal set of generators) because it is a convex combination of ***v***^2^ = (2,2,0)^*T*^ and ***v***^3^ = (2,−1,3)^*T*^, namely $v^{5}=\frac{1}{3}\;v^{2}+\frac{2}{3}\;v^{3}$. This decomposition is not conformal, and ***v***^5^ is an EFV because it results as a vertex of a subpolyhedron. In fact, the flux polyhedron consists of two subpolyhedra obtained by the intersection of the entire flux polyhedron with the orthants specified by (1,1,1) and (1,−1,1). The first subpolyhedron is a triangle with vertices ***v***^1^, ***v***^2^, and ***v***^5^, and the second one is a rectangle generated by the vertices ***v***^1^, ***v***^5^, ***v***^3^, and ***v***^4^ (Fig 4). By definition, the union of the minimal generating sets (here: vertices) of the subpolyhedra forms the set of EFVs of the entire polyhedron. Furthermore, note that ***v***^3^ is not support minimal. To illustrate property (T2’), we delete reaction R2 (fix its rate to zero). Then, the resulting flux polyhedron is the line segment between ***v***^1^ = (0,0,0)^*T*^ and ***v***^5^ = (2,0,2)^*T*^, and ***v***^1^ and ***v***^5^ form the new set of EFVs. This demonstrates why ***v***^5^ is needed as EFV.

## Computation of EFVs

Obviously, to make use of EFVs in metabolic network analyses, we need algorithms to efficiently compute EFVs. Methods based on EFMs were initially limited to comparably small networks because only a few thousand EFMs could be calculated. Due to a number of algorithmic developments in recent years [11,12,13,14,15], huge progress could be made and the calculation of millions or even billions of EFMs is now possible considerably increasing the size of the networks that can be treated. The total number of EFVs depends on size and structure of the inhomogeneous constraints; there can be fewer or more EFVs than EFMs. For this reason, efficient routines for calculating EFVs are as important as for EFMs.

Urbanczik proposed an algorithm for calculating EVs by a homogenization of the polyhedron under study [18]. For the general polyhedron (Eq 10), this yields

$$\left(\begin{array}{cc} A & -b \end{array}\right)\left(\begin{array}{c} x^{\prime }\\ \lambda \end{array}\right)\geq 0\text{ with }\lambda \geq 0.$$ (14)

Eq 14 defines a general polyhedral cone as in Eq 4. Any solution (***x***′, *λ*) of Eq 14 can be mapped to a solution ***x*** of Eq 10 as follows: if *λ* = 0 then ***x*** = ***x***′ and if *λ* ≠ 0 then ***x*** = ***x***′**/***λ*. Every solution ***x*** of Eq 10 has at least one (possibly an infinite number of) corresponding solution(s) in Eq 14, hence, the solution space of the system in Eq 10 can be generated from the solutions of Eq 14. Likewise, the EVs of Eq 14 can be directly mapped to EVs of the original polyhedron (Eq 10). To calculate the EVs from Eq 14 Urbanczik used an algorithm presented in the earlier work on conversion cones [49].

Eq 14 cannot directly be handled with standard algorithms for computing EFMs, as it forms a general polyhedral cone (Eq 4), but not a flux cone (Eq 3). We therefore use a further transformation to obtain a system in which EFM algorithms can be applied to determine the EVs of the original polyhedron in Eq 10 [18]. Concretely, by additionally introducing nonnegative slack variables ***s***, the inequalities in Eq 14 can be transformed to equalities:

$$\left(\begin{array}{ccc} A & -I & -b \end{array}\right)\left(\begin{array}{c} x\prime \\ s\\ \lambda \end{array}\right)=0\;,\;\lambda \geq 0,\;s\geq 0.$$ (15)

The general polyhedral cone in Eq 15 has the form of a flux cone (Eq 3), in which inequalities occur only as nonnegativity constraints, and the support-minimal vectors (the EFMs) of this cone correspond to the EVs of the polyhedron in Eq 10. For the flux polyhedron (Eq 9), an analogous transformation results in the system

$$Dw:=\left(\begin{array}{ccc} N & 0 & 0\\ G & -I & -h \end{array}\right)\left(\begin{array}{c} r\\ s\\ \lambda \end{array}\right)=0\;,\;r_{i}\geq 0\;\text{for}\;i\in Irr,\;\lambda \geq 0,\;s\geq 0.$$ (16)

Viewing Eq 16 as a flux cone (with ***D*** as the “stoichiometric matrix”) and computing the EFMs of this cone yield the EFVs of the flux polyhedron *FP* in Eq 9. Each EFM ***w*** = (***r***, ***s***, *λ*)^*T*^ of Eq 16 corresponds to an EFV of *FP* in the following way: if *λ* = 0, then ***u*** = ***r*** is an unbounded EFV, and if *λ* ≠ 0, then ***v*** = ***r***/*λ* is a bounded EFV, cf. Eq 13. Note that a calculated unbounded EFV ***u*** is an element of the lineality space (of the flux polyhedron’s recession cone) if, in the associated vector ***w***, it holds that ***s*** = **0** and *r*_*i*_ = 0 for *i* ∈ *Irr* (otherwise it represents a ray of the flux polyhedron’s recession cone).

To summarize, the “flux cone” in Eq 16 is obtained from the flux polyhedron (Eq 9) via homogenization and introduction of slack variables. Hence, sophisticated algorithms and tools developed for EFMs (of flux cones) can be used to compute EFVs (of flux polyhedra).

## Applications of EFVs

Because the main theoretical properties of EFMs are preserved for EFVs, the same holds true for many applications. For some applications, only the support-minimal EFVs are of interest. In those cases, all EFVs whose support is a proper superset of any other EFV can be dropped prior to the analysis. This procedure has been used, for example, in [19] to find suitable (support-minimal) EFVs that indicate feasibility of growth-coupled product synthesis.

- Application (A1’): Similar to EFMs, the support-minimal EFVs can be considered as minimal functional subnetworks for the given constraints. However, whereas EFMs typically correspond to pathways and cycles, EFVs may involve more reactions than EFMs because, apart from the steady state and reversibility constraints, also inhomogeneous flux constraints must be taken into account. For this reason, EFVs may correspond to combinations of EFMs and thus of pathways or cycles. Still, the support-minimal EFVs indicate minimal (irreducible) sets of reactions required to fulfill all constraints and can thus be used to identify minimal functional subnetworks (e.g., for synthesizing a certain product) under the given constraints.
- Application (A2’) of EFMs is also possible for EFVs: Essential, blocked, or coupled reactions can be identified via EFVs. A reaction is essential if it occurs in all (nonzero) bounded EFVs or, in case no such EFV exists, if it occurs in all unbounded EFVs. A reaction is blocked if it has zero rate in *all* EFVs. A pair of reactions is fully coupled if for each EFV either both reactions have zero rate or both reactions have nonzero rate with a constant flux ratio in all EFVs. Also, pairs of partially coupled reactions (same as for fully coupled reactions but a constant flux ratio is not required) and directionally coupled reactions (if, in each EFV, a nonzero rate of the first reaction implies a nonzero rate of the second reaction) [50] can be identified with EFVs.
- Application (A3’) of EFMs (finding optimal flux vectors) gets a largely extended scope for EFVs. Because EFMs are unbounded, EFM analysis can only deal with relative flux relationships (yields). In contrast, EFVs of a bounded flux polyhedron allow the direct consideration and analysis of both (optimal) yields and optimal (absolute) reaction rates. The optimization of a single *reaction rate* (or of a linear combination of fluxes) is expressed by a linear objective function ***c***^*T*^***r*** that is to be maximized or minimized within the flux polyhedron. If it is bounded, we know from the theory of linear programming that the optimal value is attained at a vertex (extreme point), that is, at a bounded EFV. The optimum can be immediately identified by testing which EFV maximizes the objective function. If multiple optimal solutions exist, then the space of optimal solutions forms a (sub-) polyhedron generated by the optimal bounded EFVs. For a linear optimization problem over an unbounded polyhedron with a bounded optimum, the set of optimal solutions forms again a subpolyhedron, generated by the optimal bounded EFVs *together* with all unbounded EFVs. Furthermore, a linear optimization problem with an unbounded optimal value can only arise if the objective function is unbounded on the recession cone. This occurs if the product of an unbounded EFV with the linear objective function yields a positive (in case of maximization) or negative (in case of minimization) value.
  To summarize, the set of EFVs contains *all* qualitatively distinct optimal solutions for *all possible* linear objective functions (with bounded optimum), and the solution space of a given linear optimization problem is generated by a subset of certain EFVs. Thus, EFVs allow for a comprehensive analysis of solution spaces arising in FBA optimization problems which is not possible with standard FBA techniques alone.
  **Example**: In the flux polyhedron shown in Fig 4, if we aim to maximize synthesis of product P (i.e., the rate of reaction R2), it is easy to see that the optimal solution is given by ***v***^2^, whereas ***v***^3^ is the optimal solution for maximizing the production rate of Q. Note that the latter solution does not correspond to an EFM. If the rate of reaction R1 is to be maximized, we see that three optimal EFVs exist (***v***^2^, ***v***^3^, ***v***^5^) spanning the optimal solution space for this objective function. Note again that ***v***^5^ would not be needed to span the optimal subpolyhedron; however, it represents a support-minimal and, therefore, a qualitatively distinct optimal solution. Without ***v***^5^, the remaining two EFVs could suggest that reaction R2 is essential for obtaining a maximal solution for R1, which is clearly not the case.
  Importantly, similar results follow for the maximization of *yields*, i.e., for the optimal ratio of two reaction rates *r*_*i*_*/r*_*j*_ or, more generally, for optimal ratios of linear combinations of fluxes: ***c***^*T*^***r***/***d***^*T*^***r***. From the theory of linear-fractional programming [51], we can conclude that the maximum of this objective function over a bounded flux polyhedron *FP* (with ***d***^*T*^***r*** > 0 for ***r*** ∈ *FP*) is attained at an extreme point, hence, at a bounded EFV. If multiple yield-optimal solutions exist, then, as for optimal reaction rates, the space of yield-optimal solutions forms a (sub-) polyhedron generated by the optimal bounded EFVs. In the next section, we will see that maximal synthesis rates and maximal yields of certain products are sometimes but not always attained at the same optimal EFV(s).
  If the flux polyhedron *FP* (with ***d***^*T*^***r*** > 0 for ***r*** ∈ *FP*) is unbounded, but the maximum yield (per EFV) is attained at a bounded EFV, then the yield-optimal subpolyhedron is again generated by the optimal EFVs, which may also comprise unbounded EFVs. On the other hand, if the maximum yield per EFV is attained at an unbounded EFV, then this maximum yield is not attained within the flux polyhedron. It can only be approached by making the contribution of the optimal unbounded EFV arbitrarily large. To illustrate the latter case, we set the inhomogeneous constraint *r*_2_ ≥ 1 in our example network (Fig 1). The resulting flux polyhedron has a bounded EFV ***v*** = (1,1,0)^*T*^ and an unbounded EFV ***u*** = (1,1,0)^*T*^. We might be interested in the yield of Q per used substrate S (quantified by the ratio *r*_3_/*r*_1_). Regarding the two EFVs, the maximum yield is attained at the unbounded EFV ***u***, for which *r*_3_/*r*_1_ = 1. However, within the flux polyhedron ***v*** + *β****u***. the maximum yield 1 can only be approached for *β* → *∞*, that is, for large *r*_1_ and *r*_3_.
  For all cases discussed above, if the flux polyhedron *FP* contains the zero vector and if ***d***^*T*^***r*** > 0 for ***r*** ∈ *FP* (except for ***r* = 0**), then the zero vector is a vertex of the *FP* and the yield-optimal subpolyhedron is generated by the yield-optimal EFVs and the zero vector. In particular, this includes the case of the flux cone (see application [A3]) if the restriction ***d***^*T*^***r*** > 0 for ***r*** ∈ *FP* (except for ***r*** = **0**) holds true. A detailed treatment of yield optimization on flux polyhedra is currently being prepared by some of the authors.
- Application (A4’): As EFMs, EFVs can analogously be used for computational strain and metabolic network design: due to property (T3), the effects of reaction knockouts can directly be predicted using EFVs and tailored intervention strategies blocking undesired while preserving desired phenotypes can be identified. In particular, as already pointed out in [19], (constrained) minimal cut sets can be determined from EFVs in exactly the same manner (and with the same algorithm) as used for EFMs in flux cones [37,52]. This allows, for instance, the calculation of metabolic engineering strategies in microorganisms that couple growth with product synthesis. For these calculations, it is again sufficient to focus on the support-minimal EFVs.
- Application (A5’): As for EFMs, any flux distribution within the flux polyhedron can be decomposed into EFVs, cf. Eq 13. The decomposition is again, in general, not unique and one may use similar decomposition heuristics as used in EFM-related studies. However, the interpretation might be less straightforward compared to the case of EFMs, because EFVs often involve more reactions than EFMs (see [A1’]).

Because a full enumeration of EFMs and EFVs is normally not feasible in genome-scale metabolic networks, the applications mentioned above are usually restricted to medium-scale networks, for example, to models of the central metabolism of the organisms under study. However, we believe that for a thorough understanding of constraint-based analysis techniques (often operating on flux polyhedra) one has to be aware of the notion and properties of EFVs and that these distinguished flux vectors capture key properties of the whole system. Furthermore, a number of recent studies have demonstrated that a particular subset of EFMs (e.g., the shortest EFMs [53] or EFMs involving certain reactions [54]) can be enumerated also in genome-scale networks and similar applications will also be feasible with EFVs. Finally, Kelk et al. [25] have shown that minimal generating sets of flux polyhedra spanning optimal solution spaces of FBA problems can be computed in genome-scale models. Although a minimal generating set contains only a subset of the EFVs, we anticipate that computation of all EFVs of optimal solution spaces will be feasible as well.

## Example: EFMs and EFVs in a model of the central metabolism in *Escherichia coli*

In this section, we analyze and compare sets of EFMs and EFVs in a real-world example and exemplify applications of EFVs. We used a slightly modified version of the model of the central metabolism of *E*. *coli* published in [55], which we extended by an export reaction for lysine as a biotechnologically relevant product. In the analysis, we were particularly interested in optimal production (in terms of yield *and* maximal synthesis rates) for biomass, acetate, and lysine.

We first calculated the EFMs for this network (without any flux bounds) and then the EFVs after setting an upper bound for the substrate (glucose) uptake rate (10 mmol/gDW/h) and a lower flux bound (8.39 mmol/gDW/h) for the reaction consuming ATP for nongrowth-associated maintenance processes (we denote this reaction as ATPmaint). The latter two constraints are normally used in conjunction with flux optimizations (FBA) and transform the flux cone into a flux polyhedron. Key properties of the flux cone, for example, reaction essentialities or maximal product yields as determined by EFM analysis, may not be valid anymore for the obtained flux polyhedron and requires now analysis of EFVs.

**Fig 6:**
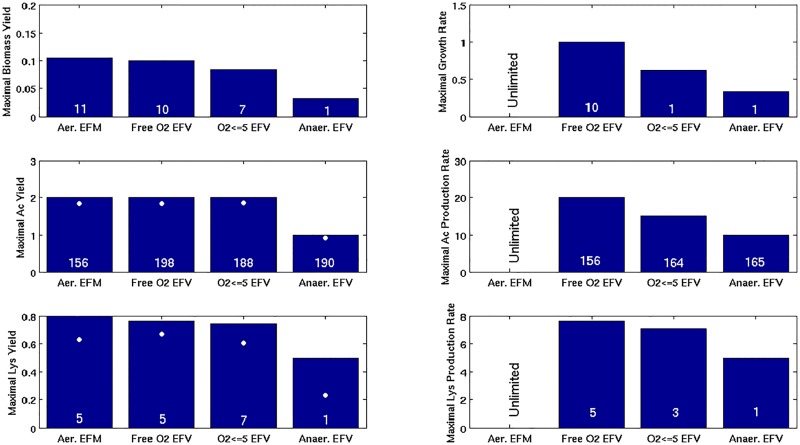
Comparison of Elementary Flux Modes (EFMs) (no flux bounds) and of Elementary Flux Vectors (EFVs) obtained by setting inhomogeneous flux constraints. The latter include (i) maximal substrate (glucose) uptake rate, (ii) ATP maintenance demand, and (iii) certain levels of oxygen availability. Glucose (Glc) was used as substrate in all scenarios. (a) maximal biomass yield (gDW/mmol Glc); (b) maximal growth-rate (h^-1^); (c) maximal acetate yield (mmol/mmol Glc); (d) maximal acetate production rate (mmol/gDW/h); (e) maximal lysine yield (mmol/mmol Glc); and (f) maximal lysine production rate (mmol/gDW/h). Maximal production rates are not given for the EFMs because EFMs can be scaled to infinity. The number of optimal EFMs/EFVs is displayed in each bar. The white circles in (c) and (e) represent the maximal guaranteed product yields for growth-coupled product synthesis (minimal demanded biomass yield is 0.01 gDW/mmol Glc).

Using the *CellNetAnalyzer* toolbox [56], we calculated 314,241 EFMs for the flux cone and 247,947 EFVs for the flux polyhedron. Thus, in this particular case, there are fewer EFVs than EFMs. All EFVs are bounded, hence, the flux polyhedron is bounded as well, in contrast to the unbounded flux cone spanned by the EFMs. The set of EFVs contains 27,864 support-minimal patterns (no EFV uses a proper subset of the reactions of any of these EFVs), thus, quite a large fraction of the EFVs is not support minimal and can be neglected for certain analyses (e.g., for reaction essentialities or computation of cut sets, but not for finding optimal flux vectors). Furthermore, because a minimum flux has been demanded for the ATPmaint reaction, the zero vector is not part of the flux polyhedron and therefore not contained in the set of EFVs. Reaction essentialities as indicated by the EFMs and EFVs, respectively, are almost identical, except that the ATPmaint reaction is contained in all bounded EFVs, and therefore, as expected, essential for all flux distribution in the polyhedron (but not in the cone). The maximal biomass yield decreases from 0.10448 gDW/mmol glucose (achievable by 11 biomass-yield optimal EFMs) in the cone to 0.10002 gDW/mmol glucose (achievable by 10 biomass-yield optimal EFVs) in the inhomogeneous case (Fig 6). In contrast, the maximum possible yield of acetate (2 mmol/mmol glucose exhibited by 198 EFVs) and of other (native) byproducts contained in the model (formate, succinate, lactate, and ethanol) did not change when introducing the constraints for glucose uptake and ATPmaint. However, we found that the maximum lysine yield reduced from 0.79730 mmol/mmol glucose in the EFMs to 0.76328 mmol/mmol glucose in the EFVs (5 yield-optimal EFVs exist). Importantly, for growth and lysine synthesis we found that the yield-optimal EFVs also correspond to the respective rate-optimal EFVs. Hence, with a maximal glucose uptake rate of 10 mmol/gDW/h, the maximal growth rate (biomass) and maximal production rate for lysine in the respective rate-optimal EFVs are 1.0002 h^-1^ and 7.6328 mmol/gDW/h, respectively. In case of acetate, we found that 156 out of the 198 yield-optimal EFVs are also rate-optimal for acetate synthesis (20 mmol/gDW/h), whereas the remaining 42 yield-optimal EFVs involve lower substrate uptake rates and hence also lowered acetate production rates.

Next, we used EFVs to characterize the impact of oxygen-limiting constraints in addition to the constraints for glucose uptake rate and ATPmaint. We set the upper boundary for the (previously unbounded) oxygen uptake reaction to 5 mmol/gDW/h resulting in 316,150 EFVs. As was highlighted above, EFVs can be used to identify both all yield-optimal as well as all rate-optimal solutions for the production of certain compounds. We therefore selected again all yield-optimal and all rate-optimal solutions for the production of biomass, acetate, and lysine (see Fig 6). As expected, for biomass we found that the maximal yield decreases compared to unlimited respiration (0.084 gDW/mmol glucose exhibited by 7 optimal EFVs). The same can be seen for the maximal growth rate (0.618 h^-1^ displayed by one EFV); however, this time biomass-yield optimal and growth-rate optimal EFVs really differ. The yield-optimal EFVs consume glucose only up to the maximum level where fully respiratory growth is possible (i.e., where the maximum uptake of oxygen is reached). In contrast, growth-rate optimal EFVs consume the maximum amount of glucose, which requires the activation of fermentative routes lowering the biomass yield but still maximizing the growth rate. Similar results can be seen for acetate (optimal yield of 2 mmol/mmol glucose [188 EFVs] and maximal production rate of 15 mmol/gDW/h [164 EFVs]) and for lysine (optimal yield of 0.743 mmol/mmol glucose [7 EFVs] and maximal production rate of 7.085 mmol/gDW/h [3 EFVs]). Interestingly, we note that the number of EFVs under moderate oxygen limitation is now larger than the number of EFMs under aerobic conditions.

When switching to fully anaerobic growth (oxygen uptake is zero) we obtain, as expected (and similar to the EFM analysis), much fewer EFVs (45,372) and reduced yields and maximal production rates for biomass, acetate and lysine (Fig 6). In this case, a single EFV is optimal for both biomass yield and growth rate and the same can be seen for maximal lysine yield and production rate. For acetate, the situation is again somewhat more complex, there are 190 yield-optimal EFVs (1 mmol/mmol glucose),165 of which are also rate-optimal (10 mmol/gDW/h).

The demonstrated analysis of yield- and rate-optimal solutions underlines one great advantage of EFVs that would, for example, not be possible with standard FBA techniques. In particular, because standard FBA problems maximize certain reaction rates, yield-space optimal solutions are usually not identified (except if they coincide with the rate-optimal solutions). Also, a full enumeration of the respective optimal EFVs allows one to find all optimal flux distributions and pathways and to get insights in properties of optimal solutions spaces. Usually, flux variability analysis is used to characterize this space, e.g., to identify which reactions are essential/exchangeable/not involved in optimal flux distributions. However, this analysis cannot extract all relevant properties, for instance, which (e.g., minimal) *combinations* of reactions yield optimal flux vectors. Another kind of subtle information that can only be obtained at the level of EFVs is feasibility of growth-coupled product synthesis, including the corresponding maximal guaranteed product yields under coupling [19]. The latter are indicated for lysine and acetate (for the different scenarios) in Fig 6 (a minimal biomass yield of 0.01 gDW /mol glucose was assumed).

We also emphasize that EFVs allow for easy implementation of thermodynamic feasibility conditions. For example, as in our case study, any EFV not involving substrate uptake correspond to thermodynamically infeasible flux distribution arising from the unbounded generators and can be eliminated. Furthermore, thermodynamic feasible EFVs can be easily obtained by the imposition of additional constraints derived from Gibbs free energy of the reactions as shown in a previous study involving EFMs [43,44,46].

## Conclusions

EFM analysis is an established tool to explore the space of stationary flux distributions in metabolic networks. However, EFMs cannot account for inhomogeneous constraints, such as known flux bounds or flux values normally used in the context of FBA. A first attempt to cope with inhomogeneous flux constraints was presented in [57]; however, this approach focused on the very special case in which all reactions in the network are irreversible and in which some fluxes are fixed to specific values (other constraints such as flux bounds were not considered). In order to generalize EFMs from flux cones to flux polyhedra, the concept of EFVs was proposed by Urbanczik one decade ago [18]. So far it has attracted much less attention than EFMs, possibly because the concept seems, at a first glance, to be more involved, although the main theoretical properties of EFMs are shared by EFVs. Moreover, apart from some specific uses, the whole spectrum of potential applications of EFVs has not been clearly communicated so far. The goal of the present work was to increase the awareness about EFVs. We explained the mathematical background of EFVs, emphasized the close relationships between EFMs and EFVs, and highlighted that almost all applications of EFMs are, in an analogous manner, possible with EFVs in flux polyhedra (partially with an even extended scope). EFVs close the gap between EFM analyses (operating on the flux cone) and FBA and related optimization techniques (usually operating on a flux polyhedron due to inhomogeneous constraints). Another reason for the so far limited use of EFVs by the community might be that their computation seemed to be less straightforward. However, it could be shown that EFVs can be calculated by well-established algorithms for computing EFMs, and this obstacle is not present anymore which should boost the applicability of the approach.

As was emphasized herein, support minimality, often considered as defining property of EFMs, is only partially preserved by the EFVs (the set of EFVs contains all minimal [support] patterns of flux vectors but possibly also nonsupport-minimal vectors). As it turns out, the key theoretical property shared by both EFMs and EFVs is (T3) / (T3’) stating that every element of the flux cone/flux polyhedron can be written as a sum of EFMs/EFVs without cancellations.

## References

[1] Lewis NE, Nagarajan H, Palsson BØ (2012) Constraining the metabolic genotype-phenotype relationship using a phylogeny of in silico methods. Nature Reviews Microbiology 10: 291–305. 10.1038/nrmicro2737 22367118 PMC3536058

[2] McCloskey D, Palsson BØ, Feist AM (2013) Basic and applied uses of genome-scale metabolic network reconstructions of Escherichia coli. Molecular Systems Biology 9: 661 10.1038/msb.2013.18 23632383 PMC3658273

[3] Klamt S, Hädicke O, von Kamp A (2014) Stoichiometric and Constraint-Based Analysis of Biochemical Reaction Networks In: Benner P, Findeisen R, Flockerzi D, Reichl U, Sundmacher K, editors. Large-Scale Networks in Engineering and Life Sciences, pp. 263–316.

[4] O'Brien EJ, Monk JM, Palsson BØ (2015) Using genome-scale models to predict biological capabilities. Cell 161: 971–987. 10.1016/j.cell.2015.05.019 26000478 PMC4451052

[5] Price ND, Reed JL, Palsson BØ (2004) Genome-scale models of microbial cells: evaluating the consequences of constraints. Nat Rev Microbiol 2: 886–897. 10.1038/nrmicro1023 15494745

[6] Clarke B (1980) Stability of complex reaction networks In: Prigogine I, Rice S, editors. Advances in Chemical Physics, Vol. 43. Wiley, New York, p. 1–216.

[7] Schuster S, Hilgetag C (1994) On elementary flux modes in biochemical reaction systems at steady state. J Biol Syst 2: 165–182.

[8] Trinh CT, Wlaschin A, Srienc F (2009) Elementary mode analysis: a useful metabolic pathway analysis tool for characterizing cellular metabolism. Applied and Environmental Microbiology 81: 813–826. 10.1007/s00253-008-1770-1 PMC2909134 19015845

[9] Zanghellini J, Ruckerbauer DE, Hanscho M, Jungreuthmayer C (2013) Elementary flux modes in a nutshell: Properties, calculation and applications. Biotechnology Journal 8: 1009–1016. 10.1002/biot.201200269 23788432

[10] Behre J, de Figueiredo LF, Schuster S, Kaleta C (2012) Detecting structural invariants in biological reaction networks. Meth Mol Biol 804: 377–407 10.1007/978-1-61779-361-5_20 22144164

[11] Terzer M, Stelling J (2008) Large-scale computation of elementary flux modes with bit pattern trees. Bioinformatics 24: 2229–2235. 10.1093/bioinformatics/btn401 18676417

[12] Urbanczik R, Wagner C (2005) An improved algorithm for stoichiometric network analysis: theory and applications. Bioinformatics 21: 1203–1210. 10.1093/bioinformatics/bti127 15539452

[13] Gagneur J, Klamt S (2004) Computation of elementary modes: a unifying framework and the new binary approach. BMC Bioinformatics 5: 175 10.1186/1471-2105-5-175 15527509 PMC544875

[14] Hunt KA, Folsom JP, Taffs RL, Carlson RP (2014) Complete enumeration of elementary flux modes through scalable demand-based subnetwork definition. Bioinformatics 30: 1569–1578. 10.1093/bioinformatics/btu021 24497502 PMC4029027

[15] van Klinken JB, Willems van Dijk K (2016) FluxModeCalculator: an efficient tool for large-scale flux mode computation. Bioinformatics 32: 1265–1266. 10.1093/bioinformatics/btv742 26685305

[16] Jungreuthmayer C, Ruckerbauer DE, Gerstl MP, Hanscho M, Zanghellini J (2015) Avoiding the enumeration of infeasible elementary flux modes by including transcriptional regulatory rules in the enumeration process saves computational costs. PLoS ONE 10: e0129840 10.1371/journal.pone.0129840 26091045 PMC4475075

[17] Orth JD, Thiele I, Palsson BØ (2010) What is flux balance analysis? Nature Biotechnology 28: 245–248. 10.1038/nbt.1614 20212490 PMC3108565

[18] Urbanczik R (2007) Enumerating constrained elementary flux vectors of metabolic networks. IET Systems Biology 1: 274–279. 17907675 10.1049/iet-syb:20060073

[19] Klamt S, Mahadevan R (2015) On the feasibility of growth-coupled product synthesis in microbial strains. Metabolic Engineering 30: 166–178. 10.1016/j.ymben.2015.05.006 26112955

[20] Müller S, Regensburger G (2016) Elementary vectors and conformal sums in polyhedral geometry and their relevance for metabolic pathway analysis. Frontiers in Genetics 7: 90 10.3389/fgene.2016.00090 27252734 PMC4877377

[21] Müller S, Regensburger G, Steuer R (2014) Enzyme allocation problems in kinetic metabolic networks: optimal solutions are elementary flux modes. Journal of Theoretical Biology 347, 182–190. 10.1016/j.jtbi.2013.11.015 24295962

[22] Schuster S, Boley D, Möller P, Stark H, Kaleta C (2015) Mathematical models for explaining the Warburg effect: a review focussed on ATP and biomass production. Biochem Soc Trans 43: 1187–1194. 10.1042/BST20150153 26614659

[23] Ziegler GM (1995) Lectures on Polytopes. Springer, New York.

[24] Bertsimas D, Tsitsiklis JN (1997) Linear optimization. Athena scientific, Belmont, Massachusetts.

[25] Kelk SM, Olivier BG, Stougie L, Bruggemann FJ (2012) Optimal flux spaces of genome-scale stoichiometric models are determined by a few subnetworks. Scientific Reports 2: 580 10.1038/srep00580 22896812 PMC3419370

[26] Schilling CH, Letscher D, Palsson BØ (2000) Theory for the systemic definition of metabolic pathways and their use in interpreting metabolic function from a pathway-oriented perspective. Journal of Theoretical Biology 203: 229–248. 10.1006/jtbi.2000.1073 10716907

[27] Klamt S and Stelling J (2003) Two approaches for metabolic pathway analysis? Trends in Biotechnology 21(2):64–69. 12573854 10.1016/s0167-7799(02)00034-3

[28] Papin JA, Stelling J, Price ND, Klamt S, Schuster S, Palsson BØ (2004) Comparison of network-based pathway analysis methods. Trends in Biotechnology 22:400–405. 10.1016/j.tibtech.2004.06.010 15283984

[29] Wagner C, Urbanczik R (2005) The Geometry of the Flux Cone of a Metabolic Network. Biophysical Journal, 89:3837–3845. 10.1529/biophysj.104.055129 16183876 PMC1366950

[30] Llaneras F, Picó J (2010) Which Metabolic Pathways Generate and Characterize the Flux Space? A Comparison among ElementaryModes, Extreme Pathways and Minimal Generators. Journal of Biomedicine and Biotechnology, 753904, 13 p. 10.1155/2010/753904 PMC2868190 20467567

[31] Carlson RP, Taffs RL (2010) Molecular-level tradeoffs and metabolic adaptation to simultaneous stressors. Current Opinion in Biotechnology 21: 670–676. 10.1016/j.copbio.2010.05.011 20637598 PMC2952661

[32] Ruckerbauer DE, Jungreuthmayer C, Zanghellini J (2015) Predicting genetic engineering targets with Elementary Flux Mode Analysis: a review of four current methods. N Biotechnol 32: 534–546. 10.1016/j.nbt.2015.03.017 25917465

[33] Maia P, Rocha M, Rocha I (2015) In Silico Constraint-Based Strain Optimization Methods: the Quest for Optimal Cell Factories. Microbiol Mol Biol Rev 80: 45–67. 10.1128/MMBR.00014-15 26609052 PMC4711187

[34] Machado D, Herrgard MJ (2015) Co-evolution of strain design methods based on flux balance and elementary mode analysis. Metabolic Engineering Communications 2: 85–92. 10.1016/j.meteno.2015.04.001 PMC8193246 34150512

[35] Trinh CT, Unrean P, Srienc F (2008) Minimal Escherichia coli cell for the most efficient production of ethanol from hexoses and pentoses. Applied and Environmental Microbiology 74: 3634–3643. 10.1128/AEM.02708-07 18424547 PMC2446564

[36] Melzer G, Esfandabadi ME, Franco-Lara E, Wittmann C (2009) Flux design: in silico design of cell factories based on correlation of pathway fluxes to desired properties. BMC Syst Biol 3: 120 10.1186/1752-0509-3-120 20035624 PMC2808316

[37] Hädicke O, Klamt S (2011) Computing complex metabolic intervention strategies using constrained minimal cut sets. Metabolic Engineering 13, 204–213. 10.1016/j.ymben.2010.12.004 21147248

[38] von Kamp A, Klamt S (2014) Enumeration of smallest intervention strategies in genome-scale metabolic networks. PLoS Comput Biol 10: e1003378 10.1371/journal.pcbi.1003378 24391481 PMC3879096

[39] Poolman MG, Venkatesh KV, Pidcock MK, Fell DA (2004) A method for the determination of flux in elementary modes, and its application to Lactobacillus rhamnosus. Biotechnology and Bioengineering 88: 601–612. 10.1002/bit.20273 15470705

[40] Schwartz J-M, Kanehisa M (2006) Quantitative elementary mode analysis of metabolic pathways: the example of yeast glycolysis. BMC Bioinformatics 7:186 10.1186/1471-2105-7-186 16584566 PMC1508158

[41] Schwarz R, Musch P, von Kamp A, Engels B, Schirmer H, Schuster S, Dandekar T (2005) YANA—a software tool for analyzing flux modes, gene-expression and enzyme activities. BMC Bioinformatics 6: 135 10.1186/1471-2105-6-135 15929789 PMC1175843

[42] Rezola A, Pey J, Figueiredo LF, Podhorski A, Schuster S, Rubio A, Planes FJ (2013) Selection of human tissue-specific elementary flux modes using gene expression data. Bioinformatics 29: 2009–16. 10.1093/bioinformatics/btt328 23742984

[43] Gerstl MP, Ruckerbauer DE, Mattanovich D, Jungreuthmayer C, Zanghellini J (2015) Metabolomics integrated elementary flux mode analysis in large metabolic networks. Scientific Reports 5: 8930 10.1038/srep08930 25754258 PMC4354105

[44] Jungreuthmayer C, Zanghellini J (2016) tEFMA: computing thermodynamically feasible elementary flux modes in metabolic networks. Bioinformatics 31: 2232–2234. 10.1093/bioinformatics/btv111 25701571

[45] Jol SJ, Kümmel A, Terzer M, Stelling J, Heinemann M (2012) System-level insights into yeast metabolism by thermodynamic analysis of elementary flux modes. PLoS Computat Biol 8: e1002415. 10.1371/journal.pcbi.1002415 PMC3296127 22416224

[46] Gerstl MP, Jungreuthmayer C, Müller S, Zanghellini J (2016) Which sets of elementary flux modes form thermodynamically feasible flux distributions? FEBS Journal 283: 1782–1794. 10.1111/febs.13702 26940826 PMC4949704

[47] Maarleveld TR, Wortel MT, Olivier BG, Teusink B, Bruggeman FJ (2015) Interplay between constraints, objectives, and optimality for genome-scale stoichiometric models. PLoS Comput Biol 11: e1004166 10.1371/journal.pcbi.1004166 25849486 PMC4388735

[48] Rockafellar RT (1969) The elementary vectors of a subspace of RN In: Combinatorial Mathematics and its Applications, Chapel Hill, University North Carolina Press, 104–127.

[49] Urbanczik R, Wagner C (2005) Functional stoichiometric analysis of metabolic networks. Bioinformatics 21: 4176–4180. 10.1093/bioinformatics/bti674 16188931

[50] Burgard AP, Nikolaev EV, Schilling CH, Maranas CD (2004) Flux coupling analysis of genome-scale metabolic network reconstructions. Genome Research 14:301–312. 10.1101/gr.1926504 14718379 PMC327106

[51] Stancu-Minasian IM (1997) Fractional programming Theory, methods and applications. Mathematics and its Applications, 409. Kluwer Academic Publishers Group, Dordrecht.

[52] Jungreuthmayer C, Nair G, Klamt S, Zanghellini J (2013) Comparison and improvement of algorithms for computing minimal cut sets. BMC Bioinformatics 14:318 10.1186/1471-2105-14-318 24191903 PMC3882775

[53] de Figueiredo LF, Podhorski A, Rubio A, Kaleta C, Beasley JE, Schuster S, Planes FJ (2009) Computing the shortest elementary flux modes in genome-scale metabolic networks. Bioinformatics 25: 3158–3165. 10.1093/bioinformatics/btp564 19793869

[54] Pey J, Planes FJ (2014). Direct calculation of elementary flux modes satisfying several biological constraints in genome-scale metabolic networks. Bioinformatics 30: 2197–2203. 10.1093/bioinformatics/btu193 24728852

[55] Hädicke O, Klamt S (2010) CASOP: a computational approach for strain optimization aiming at high productivity. Journal of Biotechnology 147: 88–101. 10.1016/j.jbiotec.2010.03.006 20303369

[56] Klamt S, Saez-Rodriguez J, Gilles ED (2007) Structural and functional analysis of cellular networks with CellNetAnalyzer. BMC Systems Biology 1: 2 10.1186/1752-0509-1-2 17408509 PMC1847467

[57] Schuster R, Schuster S (1993) Refined algorithm and computer program for calculating all non-negative fluxes admissible in steady states of biochemical reaction systems with or without some flux rates fixed. Computer applications in the biosciences: CABIOS 9: 79–85. 8435772 10.1093/bioinformatics/9.1.79

